# Self-perceptions of aging and sarcopenia in older adults: the mediating role of IADL

**DOI:** 10.3389/fmed.2025.1693158

**Published:** 2025-11-19

**Authors:** Chen Su, Sen Zhang, Qiandan Zheng, Jie Miao, Junhong Guo

**Affiliations:** 1 First Clinical Medical College, Shanxi Medical University, Taiyuan, China; 2 Department of Neurology, First Hospital, Shanxi Medical University, Taiyuan, China

**Keywords:** sarcopenia, self-perceptions of aging, instrumental activities of daily living, mediation analysis, longitudinal study

## Abstract

**Objective:**

Sarcopenia, marked by declines in muscle mass and function, poses a major risk to healthy aging. Self-perceptions of aging (SPA) reflect individuals’ attitudes toward their own aging and may influence health outcomes. This study examined the relationship between SPA and sarcopenia, focusing on the mediating role of instrumental activities of daily living (IADL).

**Methods:**

Data were drawn from adults aged 50 years and above participating in the 2014, 2016, and 2018 waves of the Health and Retirement Study. SPA was measured in 2014 using an 8-item scale. IADL were assessed in 2016 using five functional items. Sarcopenia was defined based on the revised criteria from the European Working Group on Sarcopenia in Older People. Generalized structural equation modeling estimated direct, indirect, and total effects.

**Results:**

Over 4 years, higher SPA was associated with a lower risk of sarcopenia (OR = 0.78, 95% CI: 0.65, 0.93, *p* = 0.007). Mediation analysis showed that IADL partially explained this relationship. SPA was significantly associated with grip strength, but not gait speed or muscle mass.

**Conclusion:**

IADL may serve as a behavioral pathway linking SPA to sarcopenia. Promoting positive aging beliefs and functional independence could aid in sarcopenia prevention.

## 1 Introduction

Sarcopenia, characterized by progressive losses in muscular strength and mass, is strongly associated with an increased risk of falls, functional impairment, and a diminished quality of life among older adults (1). In addition to physical decline, psychosocial determinants such as social isolation and depressive symptoms play a significant role in its development (2, 3). These factors are known to impact health behaviors, including physical activity, dietary habits, and stress management, all of which are crucial for maintaining muscle mass and function (4, 5). As the aging population grows, addressing sarcopenia has become increasingly important in public health. Identifying modifiable risk factors and effective interventions is essential.

Among the various psychosocial factors, self-perceptions of aging (SPA) have emerged as a potentially influential determinant of physical health in older individuals (6, 7). SPA encompasses individuals’ subjective attitudes and beliefs about their aging process, which can significantly affect motivation, health behaviors, and psychological resilience (8). Positive SPA is linked to increased physical activity and healthier lifestyle choices, whereas negative SPA is associated with higher levels of stress, reduced engagement in health-promoting behaviors, and an increased risk of frailty (9–11). Despite its relevance, the direct or indirect relationship between SPA and sarcopenia remains largely unexplored.

A plausible mechanism linking SPA to sarcopenia may lie in the domain of functional independence, as reflected in the performance of instrumental activities of daily living (IADL). IADL refers to higher-order daily tasks such as meal preparation, medication management, and financial handling, which require intact physical and cognitive functioning (12). IADL impairment is not only associated with frailty and cognitive decline but is also a known correlate and potential outcome of sarcopenia (13, 14). Recent studies suggest that individuals with more positive SPA are more likely to remain autonomous and engaged in these tasks, whereas negative SPA may discourage participation and reduce motivation to maintain functional ability (15, 16). These findings support the hypothesis that IADL may act as a behavioral and functional mediator between SPA and sarcopenia.

To address this gap, this study focused on assessing whether SPA predicted the incidence of sarcopenia over 4 years in a representative sample of aging adults. Additionally, this study explored IADL as a possible mediator in this link. Investigating these associations enhances our understanding of the psychosocial elements affecting sarcopenia and identifies intervention points to reduce muscle decline and enhance general health in older populations.

## 2 Methods

### 2.1 Study population

We used data from the Health and Retirement Study (HRS), a long-term national survey of Americans aged 50 years and above (17). The HRS is sponsored by the National Institute on Aging (grant number NIA U01AG009740) and is conducted by the University of Michigan. Starting in 2006, the HRS implemented the Enhanced Face-to-Face (EFTF) interview procedure, which included comprehensive social network assessments conducted in a randomly selected half-sample every 4 years. As part of the study, participants were asked to fill out the self-administered Leave-Behind Questionnaire (LBQ), which captured various aspects of psychosocial status, including social relationships, personality dimensions, lifestyle behaviors, and individual beliefs.

The HRS has received ethical approval from the institutional review board at the University of Michigan, and all participants provided informed consent prior to their participation. No additional ethical approval was required for the current study.

**Figure 1:**
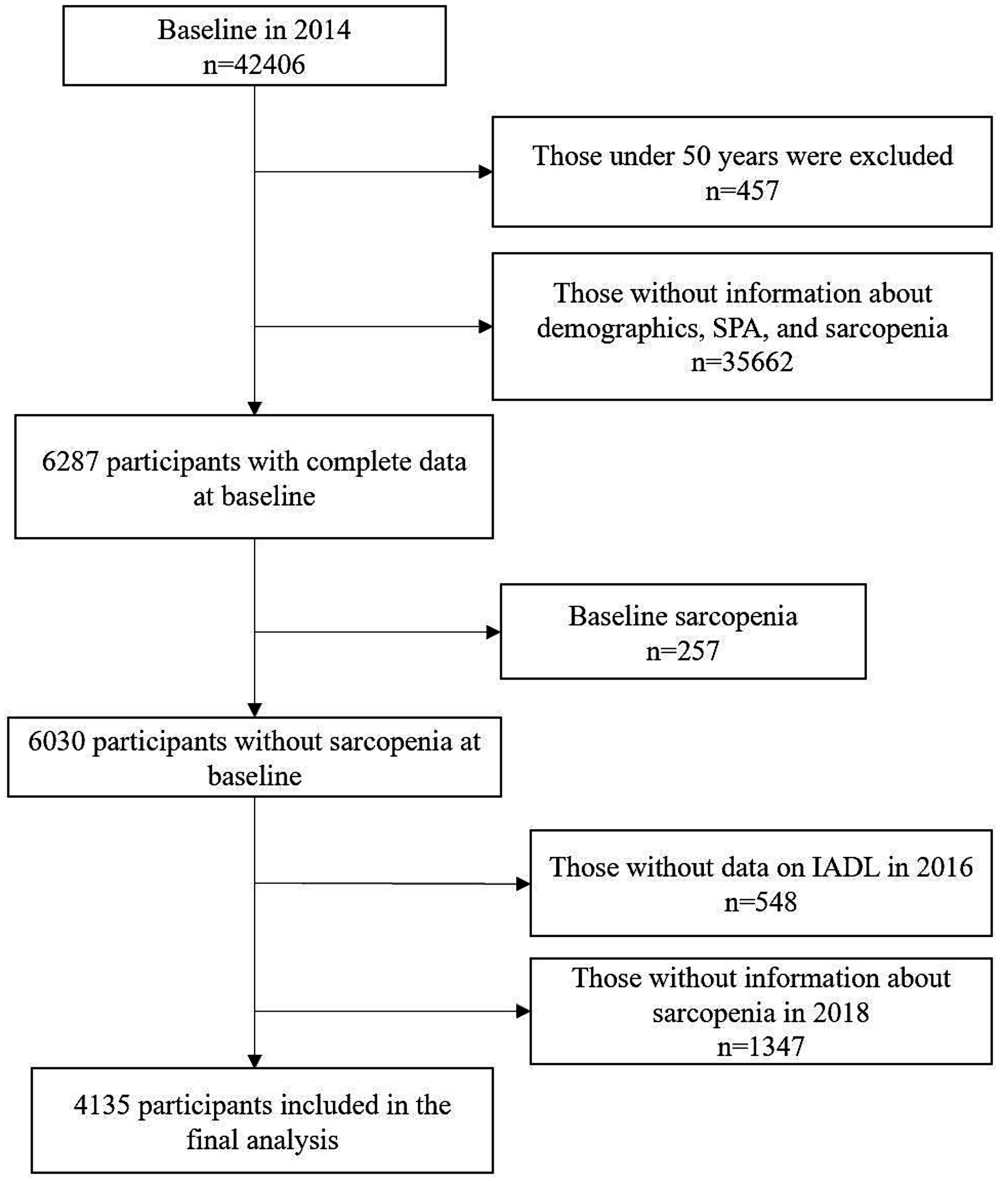
Flowchart of participants selection for the study cohort. SPA, Self-perceptions of aging.

We obtained information from the 2014, 2016, and 2018 cycles of the HRS, in combination with the RAND HRS longitudinal file (18). These datasets were merged, resulting in a pooled sample of 42,406 respondents. IADL scores were extracted from the 2016 wave to ensure temporal separation between the exposure (SPA, measured in 2014), the mediator (IADL, measured in 2016), and the outcome (sarcopenia, assessed in 2018). Participants aged 50 and above who provided complete data on demographics, SPA, IADL, muscle strength, physical function, and muscle mass were considered eligible. A flow diagram illustrating the selection process and the number of exclusions at each step is presented in Figure 1.

### 2.2 Assessment of sarcopenia

Sarcopenia classification followed the revised standards from the European Working Group on Sarcopenia in Older People (EWGSOP2) (19), incorporating three diagnostic components: (1) decreased muscular strength, evaluated by grip force cut-offs of <27 kg for men and <16 kg for women; (2) impaired physical performance, determined through gait speeds less than 0.8 m/s over a 2.5-meter course; and (3) reduced muscle quantity, quantified using appendicular skeletal muscle mass index (ASMI), with thresholds below 9.73 kg/m^2^ in females and 7.03 kg/m^2^ in males. The cut-off values for low muscle mass were derived based on the lowest sex-specific quartile of height-adjusted muscle mass within the study population (20). ASMI was estimated via a regression-based prediction model using HRS-derived parameters (21), a method that has been employed in several previous studies utilizing the HRS (3, 22). Although widely applied in previous studies, this method may not fully capture individual variations compared to direct imaging-based assessments such as dual-energy X-ray absorptiometry (DXA) or bioelectrical impedance analysis (BIA). A participant was considered sarcopenic when low handgrip strength was accompanied by reduced ASMI. When (1), (2), and (3) were all present, it was classified as severe sarcopenia.

### 2.3 Self-perceptions of aging

SPA was measured in 2014 using an 8-item scale embedded in the LBQ (23, 24). Participants responded to eight statements reflecting attitudes toward aging, including both positively and negatively framed items (e.g., “Things keep getting worse as I get older,” “I am as happy now as I was when I was younger”). Participants rated each item using a 6-point Likert scale, with values from 1 (strongly disagree) to 6 (strongly agree). Reverse scoring was applied to negatively phrased items, and an average score was computed—higher values reflected a more favorable view of aging. The internal reliability of the scale was supported by a Cronbach’s alpha value of 0.70, indicating adequate measurement consistency (25).

### 2.4 Instrumental activities of daily living

In 2016, functional status was assessed using an IADL scale included in the HRS. This scale measured participants’ ability to independently perform five instrumental daily tasks: preparing meals, managing money, taking medications, using the telephone, and shopping for groceries (12). Respondents were asked whether they had difficulty performing each task due to a health or memory problem. Each item was coded as 0 (“no difficulty”) or 1 (“some difficulty” or “unable to do”), and the total IADL score ranged from 0 to 5. Higher scores indicated greater functional impairment. This brief, validated measure has been widely used in aging research to capture early declines in daily functional independence (26).

### 2.5 Covariates

We included a range of covariates known to be associated with both sarcopenia and psychological factors. The sociodemographic covariates comprised age (as a continuous variable), gender (male or female), race (White, Black, Hispanic, or other), and education level (classified as below high school, high school, or college and above). Lifestyle factors: smoking status (ever vs. never), alcohol consumption (ever vs. never), and body mass index (BMI, calculated as kg/m^2^). Chronic health conditions: self-reported physician diagnosis of hypertension, diabetes, lung disease, heart disease, cancer, arthritis, and psychiatric conditions. All conditions were coded as binary variables (yes/no).

### 2.6 Statistical analysis

Descriptive analyses were conducted to describe the characteristics of the study cohort. Means and standard deviations were used to summarize continuous variables, whereas categorical data were described using counts and percentages. To investigate the link between SPA and sarcopenia and assess whether IADL mediate this relationship, generalized structural equation modeling (GSEM) was applied. This technique enabled concurrent estimation of both direct and indirect effects (27). In the mediation framework, SPA (from 2014) served as the independent predictor, IADL (from 2016) as the mediating variable, and sarcopenia status in 2018 as the outcome. Covariates were adjusted for in both modeling paths. To evaluate the indirect effects, bias-corrected confidence intervals (CI) were computed based on 5,000 bootstrap replications using a nonparametric approach. Statistical significance was defined as *p* < 0.05. All statistical analyses were carried out in Stata version 18.0. To confirm the stability of our results, sensitivity checks were performed. Further stratified analyses were done by gender (male/female), age group (<60 vs. ≥60), racial background, smoking behavior (ever/never), and alcohol use (ever/never). Forest plots were used to present subgroup-specific odds ratio (OR) and assess effect modification.

## 3 Results

### 3.1 Baseline characteristics

After excluding participants with missing data on key variables and those with sarcopenia at baseline, a total of 4,135 individuals were included in the final analysis (see Figure 1 for details). Among the 4,135 participants aged 50 years and older, a total of 179 individuals were diagnosed with sarcopenia. The mean age was 67.05 years, and women accounted for 59.56% of the cohort. The average BMI across the sample was 30.43 kg/m^2^. The mean scores for SPA and IADL were 4.00 and 0.16, respectively. In terms of racial composition, the majority identified as non-Hispanic White (68.7%), with smaller proportions of non-Hispanic Black (17.0%), Hispanic (11.3%), and other racial groups (3.1%). Educational attainment was distributed as follows: 12.7% had less than high school education, 60.0% completed high school, and 27.3% had college education or higher. Over half of the sample reported a history of smoking (53.1%) and alcohol use (59.0%). Common chronic conditions included arthritis (58.8%), diabetes (23.7%), hypertension (59.3%), heart disease (21.5%), psychiatric disorders (16.7%), cancer (13.9%), and lung disease (7.9%) (Table 1).

**Table 1:** Baseline characteristics of study participants.

Characteristic	Mean	SD
Age	67.05	9.16
Female, %	59.56	
BMI (kg/m^2^)	30.43	6.39
SPA	4.00	1.00
IADL	0.16	0.57
Race, %		
Non-Hispanic White	68.71	
Non-Hispanic Black	17.00	
Hispanic	11.25	
Other	3.05	
Education, %		
Below high school	12.70	
High school	59.98	
College or above	27.33	
Smoking history, %	53.08	
Drinking history, %	58.96	
Chronic disease, %		
Hypertension	59.30	
Diabetes	23.68	
Lung disease	7.91	
Heart disease	21.48	
Cancer	13.93	
Psychiatric disease	16.66	
Arthritis	58.22	

BMI, Body mass index; IADL, Instrumental activities of daily living; SD, Standard deviation; SPA, Self-perceptions of aging.

### 3.2 Association between SPA and sarcopenia

Over the four-year follow-up period, individuals reporting more favorable SPA in 2014 were less likely to develop sarcopenia by 2018. When accounting for sociodemographic characteristics, lifestyle behaviors, and health conditions in multivariate logistic regression analyses, elevated SPA scores were linked to a lower likelihood of experiencing sarcopenia (OR = 0.78, 95% CI: 0.65, 0.93, *p* = 0.007) (Table 2).

**Table 2:** The effect of SPA with sarcopenia.

Variables	OR	SE	*P*	[95% CI]
SPA	0.78	0.07	0.01	[0.65, 0.93]
Gender	0.67	0.12	0.03	[0.47, 0.96]
Race	0.77	0.11	0.07	[0.59, 1.02]
Age	1.15	0.01	<0.001	[1.12, 1.18]
BMI	0.80	0.02	<0.001	[0.77, 0.84]
Education	0.80	0.12	0.13	[0.60, 1.07]
Smoking history	1.35	0.25	0.11	[0.94, 1.93]
Drinking history	1.03	0.18	0.89	[0.72, 1.46]
Hypertension	1.44	0.28	0.06	[0.99, 2.09]
Diabetes	1.33	0.29	0.19	[0.87, 2.03]
Lung disease	1.10	0.30	0.73	[0.64, 1.88]
Heart disease	1.00	0.19	0.99	[0.69, 1.45]
Cancer	0.77	0.17	0.24	[0.50, 1.19]
Psychiatric disease	1.30	0.33	0.29	[0.80, 2.12]
Arthritis	1.47	0.29	0.05	[1.00, 2.18]

BMI, Body mass index; CI, Confidence intervals; OR, Odds ratio; SE, Standard error; SPA, Self-perceptions of aging.

### 3.3 Mediation analysis

GSEM was applied to assess whether IADL served as a mediator in the relationship between SPA and sarcopenia (Table 3). The indirect effect of SPA on sarcopenia through IADL was statistically significant (β = −0.03, 95% CI: −0.055, −0.004, *p* = 0.021). The direct path from SPA to sarcopenia also remained significant (β = −0.21, 95% CI: −0.407, −0.008, *p* = 0.041), suggesting that SPA influenced sarcopenia both directly and indirectly. The total effect was robust and statistically significant (β = −0.24, 95% CI: −0.434, −0.040, *p* = 0.018), supporting the hypothesis that IADL serves as a meaningful behavioral pathway through which SPA affects muscle health in older adults (Table 4).

**Table 3:** Mediation path model results.

Variables	IADL (β)	(SE)	Sarcopenia (β)	(SE)
Intercept	0.309^**^	0.113	−5.339^***^	1.288
Predictors				
SPA	−0.086^***^	−0.009	−0.208^*^	0.093
Mediators				
IADL			0.342^**^	0.115
Covariates				
Gender	0.004	0.018	−0.368^*^	0.185
Race	0.061^***^	0.011	−0.298^*^	0.143
Age	0.002	0.001	0.136^***^	0.011
BMI	0.001	0.001	−0.225^***^	0.022
Education	−0.062^***^	0.015	−0.191	0.150
Smoking history	0.007	0.018	0.296	0.186
Drinking history	−0.027	0.018	0.018	0.181
Hypertension	0.041^*^	0.019	0.354	0.194
Diabetes	0.018	0.021	0.282	0.217
Lung disease	0.085^**^	0.032	0.094	0.274
Heart disease	0.070^**^	0.021	−0.022	0.191
Cancer	0.039	0.025	−0.275	0.224
Psychiatric disease	0.188^***^	0.024	0.193	0.253
Arthritis	0.021	0.019	0.381	0.201

BMI, Body mass index; IADL, Instrumental activities of daily living; SE, Standard error; SPA, Self-perceptions of aging. ^*^p < 0.05; ^**^p < 0.01; ^***^p < 0.001.

**Table 4:** Parameter of mediation path.

Paths	β	SE	[95%CI]
Direct path	−0.21	0.10	[−0.407, −0.008]
Indirect path	−0.03	0.01	[−0.055, −0.004]
Total effect	−0.24	0.10	[−0.434, −0.040]

CI, Confidence intervals; SE Standard error.

### 3.4 SPA and components of sarcopenia

Further analyses explored how SPA relates to specific components of sarcopenia. Individuals with higher SPA scores exhibited a significantly lower likelihood of reduced grip strength (OR = 0.82, 95% CI: 0.73, 0.93, *p* = 0.001). However, no significant relationships were found between SPA and either slow walking speed (OR = 0.95, 95% CI: 0.87, 1.03, *p* = 0.187) or decreased muscle mass (OR = 0.93, 95% CI: 0.82, 1.04, *p* = 0.200) (Table 5).

**Table 5:** The association of SPA with sarcopenia components.

Components	OR	95% CI	*P*
Low handgrip strength	0.82	0.73, 0.93	0.001
Low gait speed	0.95	0.87, 1.03	0.197
Low ASMI	0.93	0.82, 1.04	0.200

ASMI, Appendicular skeletal muscle mass index; CI, Confidence intervals; OR, Odds ratio; SPA, Self-perceptions of aging.

### 3.5 Sensitivity analyses

**Figure 2:**
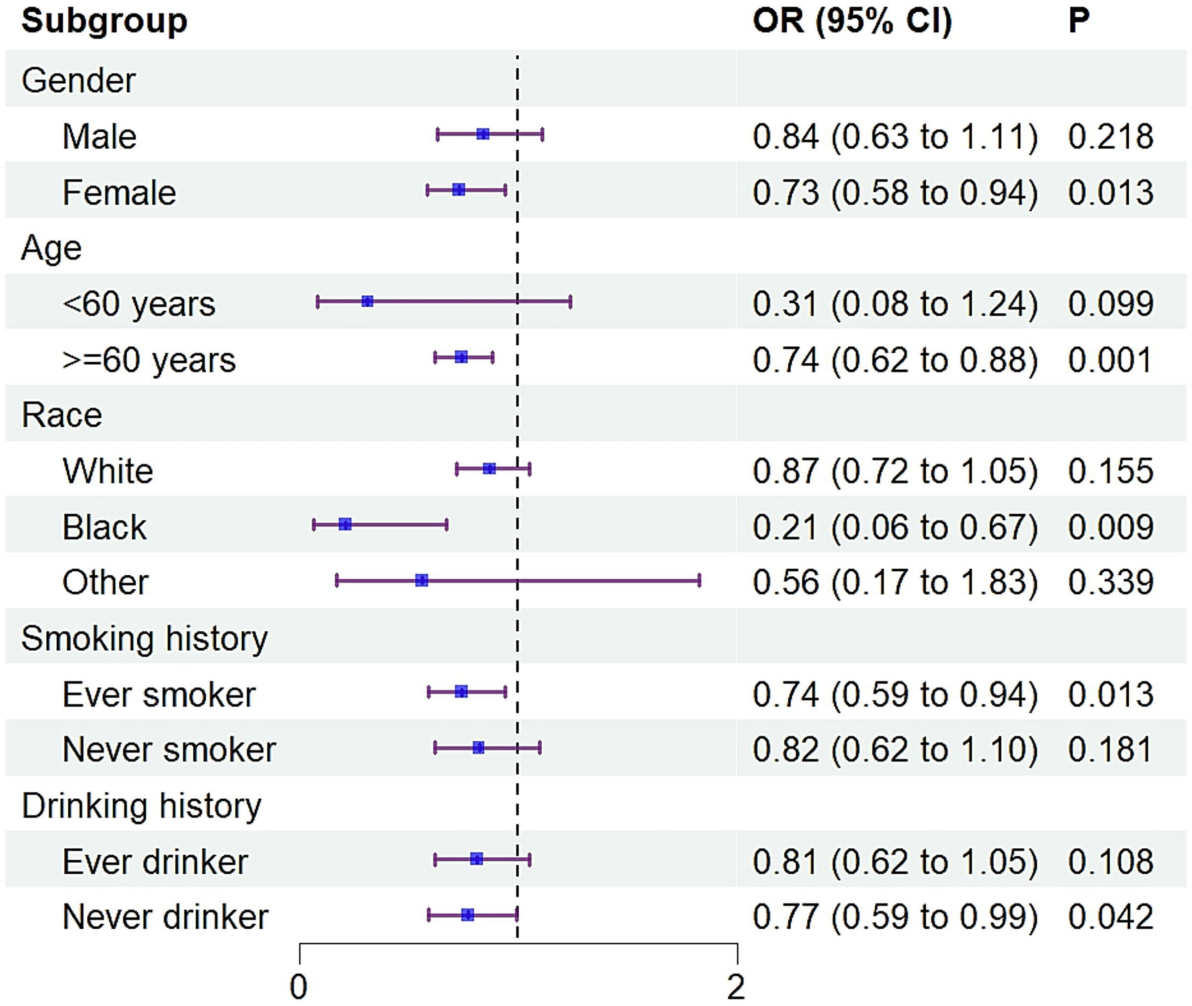
Forest plot for sensitivity analyses. CI, Confidence intervals; OR, Odds ratio.

Analyses within subgroups showed a generally consistent negative relationship between SPA and sarcopenia across different strata. Statistically meaningful associations were identified in females (OR = 0.73, 95% CI: 0.58, 0.94), individuals aged 60 and above (OR = 0.74, 95% CI: 0.62, 0.88), and among Black participants (OR = 0.21, 95% CI: 0.06, 0.67). The association also held for those with a history of smoking (OR = 0.74, 95% CI: 0.59, 0.94) and without a history of drinking (OR = 0.77, 95% CI: 0.59, 0.99). No statistically significant associations were detected in other subpopulations (Figure 2).

## 4 Discussion

To the best of our knowledge, this is the first longitudinal investigation to examine the relationship between SPA and incident sarcopenia, and to test the mediating role of IADL in a large, nationally representative cohort. After adjusting for multiple confounding factors, older adults with more positive SPA at baseline had a significantly lower risk of developing sarcopenia over 4 years. The mediation results suggested that IADL may partly explain this association, indicating a psychosocial pathway that has not been previously described.

Previous studies have shown that negative aging beliefs increase the risk of cardiovascular disease, cognitive decline, physical frailty, and mortality (9, 28, 29). Our findings extended this evidence by demonstrating that SPA also predicted sarcopenia. Previous work has focused mainly on physical activity or frailty (30, 31). In contrast, our study linked subjective aging to clinically assessed sarcopenia, highlighting SPA as a psychosocial factor relevant for prevention. This aligns with recent multidomain frameworks emphasizing that frailty is shaped by intertwined biomedical, psychosocial, and societal processes (32), highlighting the importance of SPA within broader aging research. In addition, emerging evidence suggests that environmental determinants, such as climatic factors (33), may interact with psychosocial and functional pathways to influence musculoskeletal health, underscoring the importance of multidomain models of aging (34).

One key result is the mediating role of IADL. IADL reflects complex daily functions, such as shopping, medication use, and meal preparation (35). Decreases in IADL performance have been strongly associated with increased risks of frailty, falls, and sarcopenia through both behavioral and physiological pathways (36–38). Our findings show that more positive SPA was linked to better IADL performance. This finding aligns with previous research showing that optimistic aging beliefs promote proactive health behaviors. They also support goal-directed actions and increase motivation to maintain autonomy (15, 39). These psychological traits are often linked to higher self-efficacy and resilience. They can promote greater functional independence. As a result, they may experience a slow physical decline in later life (40). These findings support the hypothesis that IADL may serve as a behavioral pathway through which SPA exerts its protective influence against sarcopenia.

An important finding of this study is that SPA was significantly associated with grip strength but not with gait speed or muscle mass. These findings suggest that SPA may have different effects on the individual components of sarcopenia. Grip strength, as a marker of neuromuscular function, is closely linked to motivational and behavioral engagement in daily activities (41–43). Older adults with more positive perceptions of aging are more likely to maintain higher levels of physical activity and daily functional engagement, which can preferentially preserve or enhance muscle strength through regular use (11, 44). In contrast, gait speed is a complex physical performance measure that depends not only on muscle strength but also on balance, joint health, neurological control, and confidence (45–48). Positive self-perceptions alone may be insufficient to influence these multifactorial determinants without targeted mobility training or behavioral interventions. Similarly, muscle mass is largely determined by long-term metabolic and hormonal factors, and may not change significantly in response to psychological factors unless positive aging beliefs translate into sustained changes in exercise or dietary behaviors (49, 50). These findings suggest that SPA affects mainly functional strength. This domain is especially sensitive to behavioral and motivational factors. In contrast, gait speed and muscle mass appear to be less influenced by SPA. This interpretation is consistent with previous evidence highlighting that muscle strength, rather than mass, underpins physical performance and functional independence in older adults (51, 52).

In the subgroup analyses, we observed notable heterogeneity in the association between SPA and sarcopenia across demographic and behavioral subgroups. The protective effect of positive SPA appeared to be more pronounced among women, older adults, individuals with a history of smoking, and those who abstained from alcohol. These patterns suggest that physiological and behavioral contexts may modulate the impact of perceptions of aging on muscle health. For example, women and older individuals generally experience greater vulnerability to physical frailty, which may heighten their sensitivity to psychosocial factors such as aging perceptions (53). Previous studies have shown that negative aging beliefs accelerate the progression of frailty, and that positive SPA is particularly predictive of physical performance in older women (54, 55). Similarly, smoking is a well-established risk factor for sarcopenia, with smokers having a two- to threefold greater risk than nonsmokers do (56). Individuals with such behavioral risk factors may rely more heavily on positive psychological resources, such as SPA, to buffer the adverse effects of these risks (57, 58). Conversely, abstaining from alcohol may reduce physiological stressors on muscle tissue. Heavy drinking has been associated with a nearly fourfold increase in the prevalence of sarcopenia among older adults. Healthier lifestyle patterns may allow positive aging perceptions to exert stronger protective effects (59, 60). The direction of association was similar across racial groups. However, significance appeared only among Black participants. This may reflect larger effect sizes, sociocultural differences in aging perceptions, or smaller sample sizes in other groups.

While our findings are based on a large, nationally representative cohort and robust analytical methods, the observational nature of this study indicates that residual confounding cannot be fully excluded. Therefore, the observed subgroup differences, such as stronger associations among women and Black participants, should be interpreted with caution. These analyses were exploratory and may have been underpowered to detect interactions with adequate precision. Future studies with larger subgroup samples and prospective designs are warranted to validate these preliminary observations.

This study is strengthened by its large-scale, population-based cohort, longitudinal design. Importantly, the temporal ordering of SPA (2014), IADL (2016), and sarcopenia outcomes (2018) supports the plausibility of causal inference within a mediation framework. However, several limitations should be noted. First, although SPA and IADL were measured prior to the outcome, the possibility of unmeasured confounding and reverse causation cannot be fully excluded. Second, a substantial number of participants were excluded because of missing data. This was partly because only approximately half of the HRS participants underwent EFTF interviews in each wave, where physical measurements were collected, leading to a large amount of missing information on muscle strength and gait speed. In addition, missing data may have arisen from attrition and incomplete responses, which could introduce selection bias. To address this, we provide a detailed flow diagram illustrating the participant selection process. Third, the ASMI was estimated using a regression-based prediction model developed for the HRS rather than being directly measured through DXA or BIA. Although this approach has been validated and widely used in previous HRS-based studies, prediction models are inherently prone to measurement error and may misclassify individuals, particularly at the boundaries of diagnostic cutoffs. Such misclassification could attenuate associations between SPA and sarcopenia or its components, potentially explaining why significant associations were observed for muscle strength but not for muscle mass. Direct measurement methods such as DXA or BIA provide more accurate estimates of lean mass and enable a more precise diagnosis of sarcopenia. Future studies incorporating objective assessments of muscle mass are warranted to validate and extend our findings. Fourth, the IADL assessment was based on self-reported functional limitations and may be subject to reporting bias. Finally, as the study sample was drawn from the U. S. population, the findings may not be generalizable to other cultural or health care contexts.

Despite these caveats, the findings underscore the potential clinical and public health importance of SPA in the context of sarcopenia prevention. If SPA indeed influences muscle health, targeted interventions to increase positive aging beliefs could represent a novel, complementary strategy alongside conventional physical and nutritional approaches. Psychological interventions, such as cognitive–behavioral therapy and psychoeducational programs, have been shown to improve attitudes toward aging by challenging negative stereotypes and strengthening self-efficacy (61). Behavioral interventions that foster engagement in new skills, physical activity, or mindful reflection may further reinforce these effects by translating positive beliefs into sustained health behaviors (62). Community-based programs, including intergenerational activities and structured life-review groups, have also been effective in shifting aging narratives and promoting social participation (61, 63). Although no randomized trials have examined SPA-focused interventions for sarcopenia, evidence shows that positive SPA is linked to higher physical activity, better physical performance, and lower inflammation. These pathways may explain its potential impact (64, 65). Recent work using quantitative sensory testing in musculoskeletal disorders highlights the prognostic value of sensory processing pathways (66). Similarly, emerging evidence linking gut microbiome metabolites to pain modulation suggests that biological mechanisms may interact with psychosocial factors to shape musculoskeletal aging (67). Integrating these perspectives could help design more effective, multidimensional interventions. Future research should evaluate whether integrating SPA-targeted components into exercise or lifestyle interventions enhances adherence and improves muscle health outcomes. Interdisciplinary approaches that combine psychosocial, behavioral, and biological perspectives may offer new opportunities for sarcopenia prevention. In addition, integrating psychosocial markers such as SPA into predictive models of aging, including machine learning–based approaches for frailty detection, may enhance early identification and personalized prevention strategies (68).

## 5 Conclusion

This four-year prospective study of older Americans revealed that individuals with more favorable SPA exhibited a decreased risk of developing sarcopenia. IADL partially explained this association, suggesting a potential behavioral pathway through which SPA influences physical aging. These findings underscore the importance of incorporating subjective aging beliefs into sarcopenia prevention strategies and highlight the value of maintaining daily functional capacity in later life. Interventions aimed at enhancing aging perceptions and preserving IADL abilities may hold promise for mitigating age-related muscle decline.

## Data Availability

Publicly available datasets were analyzed in this study. This data can be found here: The data used in this study are publicly available from the Health and Retirement Study (HRS) at https://hrs.isr.umich.edu/.
